# Dasatinib-Associated Acneiform Eruption Successfully Treated With Sarecycline in a Patient With Chronic Myeloid Leukemia

**DOI:** 10.7759/cureus.45697

**Published:** 2023-09-21

**Authors:** Dina Poplausky, Jade N Young, Danielle P Dubin, Douglas Tremblay, Nicholas Gulati

**Affiliations:** 1 Department of Dermatology, Icahn School of Medicine at Mount Sinai, New York, USA; 2 Division of Hematology and Medical Oncology, The Tisch Cancer Institute, Icahn School of Medicine at Mount Sinai, New York, USA

**Keywords:** skin toxicity, acneiform eruptions, sarecycline, chronic myeloid leukemia, dasatinib

## Abstract

Dasatinib is a second-generation tyrosine inhibitor that is used for the treatment of patients with chronic myeloid leukemia (CML). It can cause a myriad of skin toxicities, including pruritis, pigmentary abnormalities of hair and skin, and maculopapular rashes. Rarely, it can be associated with acneiform eruptions, which are typically treated with doxycycline. However, doxycycline may not be an ideal therapy, especially for long-term use, due to the risk of gut flora disruption, antimicrobial resistance, and side effects. We present a case of a CML patient who developed an acneiform eruption associated with dasatinib and was successfully treated with sarecycline, a narrow-spectrum tetracycline. Given its targeted spectrum of activity, sarecycline has a lower risk of antimicrobial resistance and an improved safety profile compared to first- and second-generation tetracyclines such as doxycycline. As acneiform drug eruptions can have a significant impact on a patient’s quality of life, effective management by dermatologists is paramount. Sarecycline may be a suitable treatment with a favorable safety profile, making it an appropriate choice for patients, especially those who require long-term therapy.

## Introduction

Dasatinib is a second-generation tyrosine inhibitor that is used for the treatment of patients with chronic myeloid leukemia (CML) or Philadelphia chromosome-positive acute lymphoblastic leukemia (Ph+ ALL). Its mechanism of action involves the blockade of breakpoint cluster region-Abelson (BCR-ABL) and SRC family kinases. It has several advantages over imatinib, a common first-line treatment in CML, as it is more potent and patients are less likely to develop resistance to it [1]. Common skin toxicities associated with dasatinib include pruritus, pigmentary abnormalities of hair and skin, and maculopapular rash [2]. Rarely, it can be associated with acneiform eruptions [2,3]. We describe, to our knowledge, the first case of an acneiform eruption associated with dasatinib successfully treated with sarecycline, a narrow-spectrum tetracycline.

## Case presentation

A 39-year-old male with CML underwent a year of therapy with imatinib 400 mg daily. Due to a sub-optimal molecular response, he was subsequently transitioned to dasatinib 100 mg daily. One month after dasatinib initiation, the patient developed painful, erythematous papules, pustules, and cysts on his face, trunk, and upper extremities. Due to the clinical examination and temporal pattern, he was diagnosed with an acneiform eruption secondary to dasatinib use. His presentation was congruent with previously reported acneiform eruptions associated with multikinase inhibitors, which typically cause a papulopustular rash on the face and upper trunk within eight weeks of medication initiation [4]. At the time of rash onset, he was not taking any other daily medications, and no other new medications were started within at least the prior four months.

**Figure 1:**
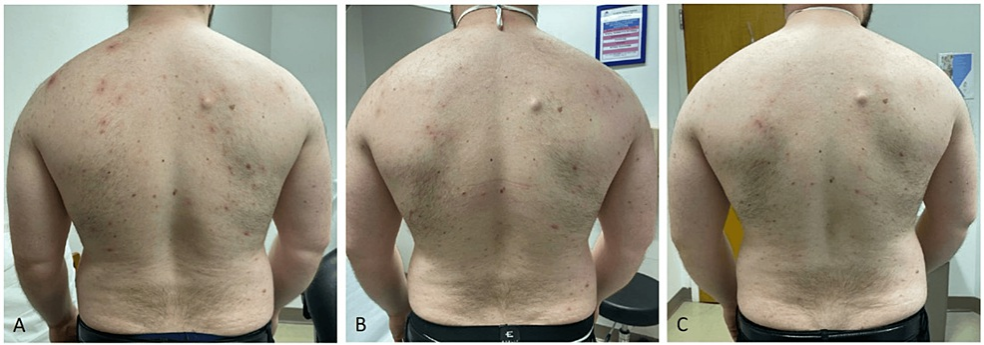
Clinical course A) Erythematous papules and pustules throughout the patient’s back, consistent with an acneiform eruption, nine months after dasatinib initiation. B) Improved acneiform eruption after a three-month course of doxycycline 100 mg twice daily. C) Sustained improvement one month after sarecycline 150 mg once daily initiation.

The patient achieved an improved molecular response with dasatinib and opted to continue the medication. Eight months after the rash onset, the patient sought dermatologic evaluation for symptomatic treatment of his cutaneous eruption. Due to the extent of the acneiform lesions and inflammation (Figure 1A), the patient was started on oral treatment with doxycycline 100 mg twice daily; after three months of treatment, the patient demonstrated marked improvement (Figure 1B). Given the side effects associated with long-term doxycycline use, including antimicrobial resistance and gastrointestinal dysfunction, the patient was transitioned to sarecycline 150 mg once daily. After one month of therapy, the patient achieved near-complete skin clearance (Figure 1C). One month later, the patient discontinued sarecycline due to insurance issues. Table 1 describes the various medications throughout his clinical course.

**Table 1:** Relevant patient medications

Medication	Dosage	Route	Frequency
Dasatinib	100 mg	Oral	Daily
Doxycycline	100 mg	Oral	Daily
Sarecycline	150 mg	Oral	Daily

One and a half months after sarecycline cessation, his acneiform eruption flared, and he was subsequently restarted on sarecycline 150 mg once daily. The patient completed a second two-month course without any noted side effects, and again achieved near-complete clearance. Thereafter, he was maintained with topical therapy only. Importantly, the patient remained on dasatinib 100 mg throughout this time course, without any oncologic therapy interruptions or dose modifications. At his most recent oncologic follow-up visit 17 months after dasatinib initiation, the patient achieved a goal molecular response. 

## Discussion

To the authors’ knowledge, this is the first report of acneiform skin toxicity associated with dasatinib that was successfully treated with sarecycline. The patient did not require any dasatinib treatment interruptions or dose adjustments. To date, there are a few cases of dasatinib-associated acneiform eruptions described in the literature, some of which were treated with doxycycline and intralesional triamcinolone injections [3]. However, doxycycline may not be the ideal therapy, especially for long-term use, due to the risk of gut flora disruption, antimicrobial resistance, and side effects [5].

Various medications have been associated with pustular dermatoses, which can be generalized or localized, such as in the case of acneiform eruptions. These typically affect the face, chest, and back [6]. Common oncologic therapies known to cause acneiform eruptions include epidermal growth factor receptor (EGFR) inhibitors and MEK inhibitors [7]. EGFR inhibitors cause epidermal keratinocytes to secrete pro-inflammatory chemokines and cytokines. MEK is a downstream molecule of the EGFR pathway and therefore the associated eruptions likely have a similar pathogenesis [7]. The mechanism of the acneiform eruption associated with tyrosine kinase inhibitors, such as dasatinib, is not well-elucidated in the literature; however, it is hypothesized to be due to a disturbance in keratinization [2].

Acneiform eruptions associated with cancer therapy can negatively impact a patient’s quality of life [8] and may lead to dose modification or drug discontinuation [9]. Therefore, adequately addressing these adverse effects is imperative so that patients may continue potentially life-saving treatment. The European Society for Medical Oncology (ESMO) generated a system to classify the severity of dermatologic toxicities and their associated management [7]. Grades 1 and 2 acneiform eruptions are typically managed with oral doxycycline or minocycline and a topical low to moderate-potency corticosteroid. Reactions Grade 3 and above are managed similarly to Grades 1 and 2 reactions but with the addition of systemic corticosteroids and/or low-dose isotretinoin. Oral antibiotic treatment duration for all grades is typically about six weeks. Preventative measures, such as prophylactic oral antibiotics, sun protective measures, and skin irritant avoidance, are also emphasized by the ESMO guidelines [7].

Sarecycline is a narrow-spectrum third-generation tetracycline that is Food and Drug Administration (FDA)-approved for the treatment of moderate-to-severe acne. Given its targeted spectrum of activity, it has a lower risk of antimicrobial resistance and an improved safety profile compared to first- and second-generation tetracyclines such as doxycycline and minocycline. A Phase III clinical trial found it to be safe and tolerable in acne patients for up to one year [5]. Thus, sarecycline is a viable long-term treatment option for patients who have partial or complete responses to doxycycline, but who have met or exceeded the recommended duration of therapy. Aside from its antimicrobial effects, sarecycline affects the Wnt and Hedgehog molecular pathways, both of which have been implicated in the pathogenesis of acne [10,11].

## Conclusions

We report a case of an acneiform eruption associated with dasatinib, which is commonly used for the treatment of CML, both first-line and after imatinib failure. As acneiform drug eruptions can have significant impacts on a patient’s quality of life, healthcare providers and patients alike must be aware of this possible side effect and its associated management. Sarecycline may be an effective treatment with a favorable safety profile, making it an appropriate choice for patients, particularly those who require long-term therapy.

## References

[1] Aguilera DG, Tsimberidou AM (2009). Dasatinib in chronic myeloid leukemia: a review. Ther Clin Risk Manag.

[2] Belum VR, Washington C, Pratilas CA, Sibaud V, Boralevi F, Lacouture ME (2015). Dermatologic adverse events in pediatric patients receiving targeted anticancer therapies: a pooled analysis. Pediatr Blood Cancer.

[3] Jung YS, Kim M, Lee JH, Kim DW, Park HJ (2016). Acneiform eruptions caused by various second-generation tyrosine kinase inhibitors in patients with chronic myeloid leukaemia. Br J Dermatol.

[4] Yuan C, Wang B (2023). Acneiform eruption induced by molecularly targeted agents in antineoplastic therapy: a review. J Cosmet Dermatol.

[5] Pariser DM, Green LJ, Lain EL, Schmitz C, Chinigo AS, McNamee B, Berk DR (2019). Safety and tolerability of sarecycline for the treatment of acne vulgaris: results from a Phase III, multicenter, open-label study and a Phase I phototoxicity study. J Clin Aesthet Dermatol.

[6] Mengesha YM, Bennett ML (2002). Pustular skin disorders: diagnosis and treatment. Am J Clin Dermatol.

[7] Lacouture ME, Sibaud V, Gerber PA (2021). Prevention and management of dermatological toxicities related to anticancer agents: ESMO Clinical Practice Guidelines☆. Ann Oncol.

[8] Joshi SS, Ortiz S, Witherspoon JN (2010). Effects of epidermal growth factor receptor inhibitor-induced dermatologic toxicities on quality of life. Cancer.

[9] Ibraheim MK, Lo J, Gupta R, Parseghian C, Patel AB (2022). Acneiform eruptions with combination targeted cancer therapy in colorectal cancer patients. Support Care Cancer.

[10] Martin V, Grenho L, Fernandes MH, Gomes PS (2023). Repurposing sarecycline for osteoinductive therapies: an in vitro and ex vivo assessment. J Bone Miner Metab.

[11] Allen M, Grachtchouk M, Sheng H (2003). Hedgehog signaling regulates sebaceous gland development. Am J Pathol.

